# Factors Predicting Prognosis in Metastatic Grade 1 Gastro-entero-pancreatic Neuroendocrine Tumors

**DOI:** 10.1007/s12029-024-01077-9

**Published:** 2024-06-14

**Authors:** Saneya A. Pandrowala, Deeksha Kapoor, Aditya Kunte, Amit Chopde, Ameya Puranik, Indraja Devidas Dev, Rahul Parghane, Sandip Basu, Anant Ramaswamy, Vikas Ostwal, Vikram A. Chaudhari, Manish S. Bhandare, Shailesh V. Shrikhande

**Affiliations:** 1 Gastrointestinal and Hepato-Pancreato-Biliary Service, Department of Surgical Oncology, Homi Bhabha National Institute, Tata Memorial Hospital, Mumbai, Maharashtra 400012 India; 2 Department of Nuclear Medicine and Molecular Imaging, Tata Memorial Centre, Homi Bhabha National Institute, Mumbai, Maharashtra 400012 India; 3 Radiation Medicine Centre, Bhabha Atomic Research Centre, Tata Memorial Hospital Annexe, Mumbai, Maharashtra 400012 India; 4 Department of Medical Oncology, Tata Memorial Centre, Homi Bhabha National Institute, Mumbai, Maharashtra 400012 India

**Keywords:** Neuroendocrine tumor, Grade 1, Metastases, Progression-free survival, Overall survival

## Abstract

**Introduction:**

The incidence of gastroenteropancreatic neuroendocrine tumors (GEP-NET) has steadily increased. These tumors are considered relatively indolent even when metastatic. What determines survival outcomes in such situations is understudied.

**Materials and Methods:**

Retrospective analysis of a prospectively maintained NET clinic database, to include patients of metastatic grade 1 GEP-NET, from January 2018 to December 2021, to assess factors affecting progression-free survival (PFS).

**Results:**

Of the 589 patients of GEP-NET treated during the study period, 100 were grade 1, with radiological evidence of distant metastasis. The median age was 50 years, with 67% being men. Of these, 15 patients were observed, while 85 patients received treatment in the form of surgery (*n* = 32), peptide receptor radionuclide therapy (*n* = 50), octreotide LAR (*n* = 22), and/or chemotherapy (*n* = 4), either as a single modality or multi-modality treatment. The median (PFS) was 54.5 months. The estimated 3-year PFS and 3-year overall survival rates were 72.3% (SE 0.048) and 93.4% (SE 0.026), respectively. On Cox regression, a high liver tumor burden was the only independent predictor of PFS (OR 3.443, *p* = 0.014). The 5-year OS of patients with concomitant extra-hepatic disease was significantly lower than that of patients with liver-limited disease (70.7% vs. 100%, *p* = 0.017).

**Conclusion:**

A higher burden of liver disease is associated with shorter PFS in patients with metastatic grade I GEP-NETs. The OS is significantly lower in patients with associated extrahepatic involvement. These parameters may justify a more aggressive treatment approach in metastatic grade 1 GEP-NETs.

## Introduction

Though gastroenteropancreatic neuroendocrine tumors (GEP-NETs) are considered rare, their incidence has steadily increased [1, 2]. This can be attributed to a better understanding of the disease, early detection because of improved imaging techniques, and possible stage migration [2]. As a result, these small arterially enhancing lesions are identified early and treated [2]. Nevertheless, the incidence of metastatic NETs has remained relatively stable, with a greater increase in the incidence of localized disease across all sites [3]. The management of GEP-NET is challenging, with multiple diverse therapeutic options available. Treatment is most importantly guided by the Ki 67 index [4]. The choice of therapy is also influenced by the tumor site, functionality, metastasis site, disease burden, performance status, and patient preference [4, 5]. Multiple management guidelines are available, confusing scenario management [6–8]. The authors believe that treatment decisions for GEP-NETs should be formulated in a multidisciplinary clinic and include the perspective of each specialist.

The involvement of regional lymph nodes and distant metastasis are considered to impact overall outcomes [9, 10]. This is reflected in the tumor, node and metastasis (TNM) staging, which was proposed by the European Neuroendocrine Tumour Society (ENETS) and was adopted by the 8th edition of UICC (Union for International Cancer Control) [11]. The management of metastatic GEP-NETs includes a myriad of options, from observation, long-acting somatostatin analogues (LAR), peptide receptor radionuclide therapy (PRRT), surgery, and even chemotherapy [12–14]. Despite stage 4 disease, a nihilistic approach to management may not be prudent in grade 1 GEP-NETS with metastasis, given their indolent nature. Which patients with metastatic grade 1 GEP-NETs fare poorly and what portends a worse prognosis in this so-called ‘indolent’ group is sparsely studied. The present study aimed to ascertain the biological behavior of grade 1 metastatic GEP-NETs and to identify factors impacting overall survival and progression-free survival in this cohort of patients.

## Materials and Methods

The present study is a retrospective analysis of a prospectively maintained database of the NET clinic at Tata Memorial Hospital, Mumbai, from January 2018 to December 2021. The study was carried out per national and international guidelines and the basic principles of protecting the rights and dignity of human beings, as set out in the Helsinki Declaration (64th Assembly Fortaleza, Brazil, in October 2013) [15]. All patients with metastatic, grade 1 GEP-NETs (Mib-1 < 2%) were included in this study. Patients with mixed neuroendocrine-non-neuroendocrine neoplasms, the syndromic occurrence of NET, and those with synchronous malignancies were excluded. For formulating the treatment plan, all patients were discussed in a dedicated multidisciplinary NET clinic, including a surgeon, medical oncologist, pathologist, and nuclear physician. A somatostatin receptor (SSTR) imaging with a positron emission tomography (SSTR–PET, with ^68^ Ga-DOTATATE) was routinely performed to assess the SSTR avidity of the tumor and for assessment of metastatic disease [14]. Without or with low SSTR uptake on the SSTR scan, an ^18^fluorodeoxyglucose (FDG) PET scan was performed for dual assessment [14]. The NET grade was confirmed by an experienced pathologist, and MiB 1 staining was used for Ki–67 assessment as a standard protocol as per the Institute. In case of suboptimal paraffin blocks, extensive disease burden, or unexplained FDG avidity, a repeat biopsy was considered from the FDG avid lesion to evaluate for grade discordance.

The management was based on the primary tumor site and the resectability of the primary and metastatic sites to achieve R0 resection and patient symptomatology. As per the imaging, a curative surgical plan was preferred if complete removal was possible. If R0 resection was not feasible, PRRT, with neoadjuvant intent, was considered, followed by surgical assessment [16, 17]. PRRT therapy was performed with radiolabelled somatostatin analogue 177-Lutetiem (^177^-Lu) DOTATE. For patients deemed unresectable, the treatment options offered were observation, LAR, PRRT, or chemotherapy, either in combination or alone, based on clinical symptoms, SSTR uptake, and FDG avidity. Capecitabine plus temozolamide was considered as the chemotherapy regimen for patients with FDG avid lesions. Patients with liver-limited disease were considered for trans-arterial embolization (TAE) or intra-arterial PRRT. Demographic variables and survival outcomes based on type of treatment were collected and analyzed. The burden of liver disease was assessed radiologically in a semi-quantitative manner and defined as high volume (> 50% of liver parenchyma) or low volume (< 50% of liver parenchyma).

### Follow-Up

Follow-up protocol included 3–6 monthly interval visits for the first 2 years and 6 monthly for 5 years without any progression, using an abdomen ultrasound, serum chromogranin A levels, and routine blood tests. During active treatment and if there was progression on imaging, a 3-month follow-up was performed. Depending on the expression pattern at baseline, SSTR imaging or FDG scan was performed every 6 months or as clinically indicated. Median overall survival (OS) was calculated from the date of diagnosis until the date of last follow-up or death. Progression-free survival (PFS) was calculated from the date of diagnosis to disease progression or from the date of surgical resection to either disease recurrence or radiological disease progression.

### Statistical Analysis

Categorical variables are expressed as proportions and continuous variables as median and interquartile ranges. Categorical variables were compared using the Pearson Chi-square test. Continuous variables were compared using a paired t-test for parametric data and Mann–Whitney *U*-test for non-parametric data. Survival analyses were performed using the Kaplan–Meier survival curves. Survival was compared by using the log-rank test. A multivariate, step-wise Cox regression was performed to assess the independent contribution of various factors on survival. A *p*-value of ≤ 0.05 was considered statistically significant. All analyses were performed on IBM SPSS Statistics for Windows, Version 25.0. Armonk, NY: IBM Corp.

## Results

### Study Population

**Fig. 1:**
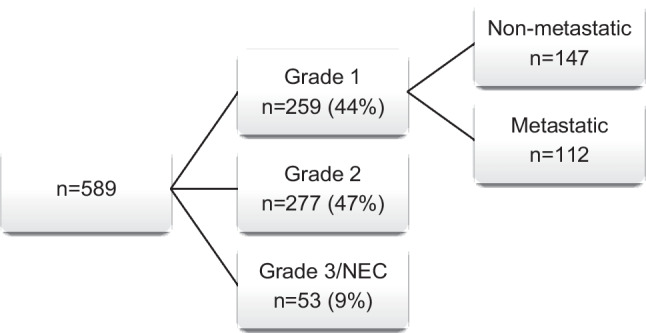
Patients seen in the NET clinic from January 2018 to December 2021

A total of 589 patients with neuroendocrine tumors were seen in the NET clinic from January 2018 to December 2021. To achieve a follow-up of at least 24 months, patients with grade 1 GEP-NETs treated till February 2021 were included (Fig. 1). The final analysis included 100 patients with metastatic, grade 1 well-differentiated GEP-NETs. The median age of the study population was 50 years (IQR 40–60 years), with 67% of patients being men. The most common site of the primary tumor was the duodeno-pancreatic complex, followed by small bowel, seen in 45% and 27% of patients, respectively. Among these, 33% were functional tumors, and 73% of patients had metastatic liver disease. In patients with liver metastasis, 46 (63%) had high-volume liver disease and 27 (37%) had low-volume liver disease. Of the entire cohort, 15 patients received no treatment for the metastatic disease and were only observed. Four (26.6%) of these patients had undergone resection of their primary tumor before presenting at our institute. The other 85 patients received treatment in the form of surgery (*n* = 32), PRRT (*n* = 50), octreotide LAR (*n* = 22), and/or chemotherapy either as a single modality or as part of multi-modality management (Table 1). The various primary treatment modalities used in the cohort are described in Table 2. Of these, 44 patients underwent liver-directed ablative therapy through transarterial embolization (17/44) and radiofrequency ablation (27/44).

**Table 1:** Clinicopathological characteristics of the study population

Variable		Total (*n* = 100)		Observation (*n* = 15)		Treated (*n* = 85)		*p*-value
		*n*	%	*n*	%	*n*	%	
**Age in years**		50 (40–60)^¶^		47 (39–56)		52 (40–60)		0.222
**Sex**	Male	57	57	11	73.3	56	65.9	0.572
	Female	33	33	4	26.7	29	34.7	
**Primary site**	Duodeno-pancratic	45	45	6	40	39	45.9	0.490
	Small bowel	27	27	4	26.7	23	27.1	
	Gastric	4	4	1	6.7	3	3.5	
	Hepato-biliary	5	5	2	13.3	3	3.5	
	Colorectal	8	8	2	13.3	6	7.1	
	Other	6	6	-	-	6	7.1	
	Unknown	5	5	-	-	5	5.9	
**Functional Status**	No	67	67	10	66.7	57	67.1	0.976
	Yes	33	33	5	33.3	28	32.9	
**Site of matastases**	Extra-hepatic	27	27	3	20.0	24	28.2	0.262
	Liver-only	29	29	7	46.7	22	25.9	
	Liver + extrahepatic	44	44	5	33.3	39	45.9	
**Median number of liver merastases**		5.5 (0-10)		8 (1-10)		5 (0-10)		0.589
**Liver disease burden**	None	27	27	3	20	24	28.2	0.747
	Low-volume	27	27	5	33.3	22	25.9	
	High-volume	46	46	1	46.7	39	45.9	
**Treatment received**	Observation	15	15	15	100	-	-	-
	LAR	15	15	-	-	15	17.6	
	LAR + PRRT	7	7	-	-	7	8.2	
	PRRT	27	27	-	-	27	31.8	
	Surgery	20	20	-	-	20	23.5	
	Surgery + PRRT	12	12	-	-	12	14.1	
	PRRT + chemo	4	4	-	-	4	4.7	
**Surgery for primary**	No	66	66	11	73.3	55	64.7	0.151
	Yes	34	34	4	26.7	30	35.3	
**Surgery for metastases**	No	68	68	15	100	53	62.4	-
	Yes	32	32	-	-	32	37.6	

Inter-quartile range in parentheses

**Table 2:** Primary treatment modalities used in the present cohort of patients

**Extent of metastatic disease**		**Treatment modality used**						
		**Observation**	**LAR**	**LAR + PRRT**	**PRRT alone**	**Surgery**	**Surgery + PRRT**	**PRRT + chemotherapy**
**Low liver burden**	*n* = 27	5	2	1	8	6	5	-
**High liver burden**	*n* = 46	7	12	6	13	1	4	3
**Only extrahepatic disease**	*n* = 27	3	1	-	6	13	3	1

### Survival Outcomes

The median duration of follow-up was 31.8 (IQR 20.45–45.29) months for the entire cohort. The median PFS was 54.5 months (95% CI 32.78–76.23 months). The entire cohort's estimated 3-year PFS and 3-year OS rates were 72.3% (SE 0.048) and 93.4% (SE 0.026), respectively.

### Factors Affecting Survival

To study PFS, various factors, including age, sex, site of the disease, functionality, burden of liver disease, and treatment options used, were studied (Table 3). Step-wise multivariate Cox regression was performed to study the independent impact of each parameter on PFS (Table 3). Of these, a high disease burden in the liver was found to be the only independent predictor of PFS (OR 3.443 (95% CI 1.288–9.205), *p* = 0.014).

**Table 3:** Univariate and multivariate regression analyses for factors affecting PFS

**Variable**	**Univariate *p*-value**	**Multivariate *p*-value**	**Odds ratio**	**95% CI**
**Age**	0.979	-	-	-
**Sex** Male Female	Ref 0.67	- -	- -	
**Primary site** Duodeno-pancreatic Esophago-gastric Small bowel Colorectal Hepato-biliary Retroperitoneal/other	Ref 0.856 0.957 0.432 0.532 0.961	- - - - - -	- - - - - -	- - - - - -
**Functional tumor** No Yes	Ref 0.100	- -	- -	- -
**Liver disease burden** None Low High	Ref 0.422 0.006	- 0.354 0.014	- - 3.443	- - 1.288 – 9.205
**Treatment received** Observation LAR PRRT Surgery	Ref 0.033 0.364 0.600	- - - -	- - - -	- - - -

### Impact of Liver Disease Burden on Survival

**Fig. 2:**
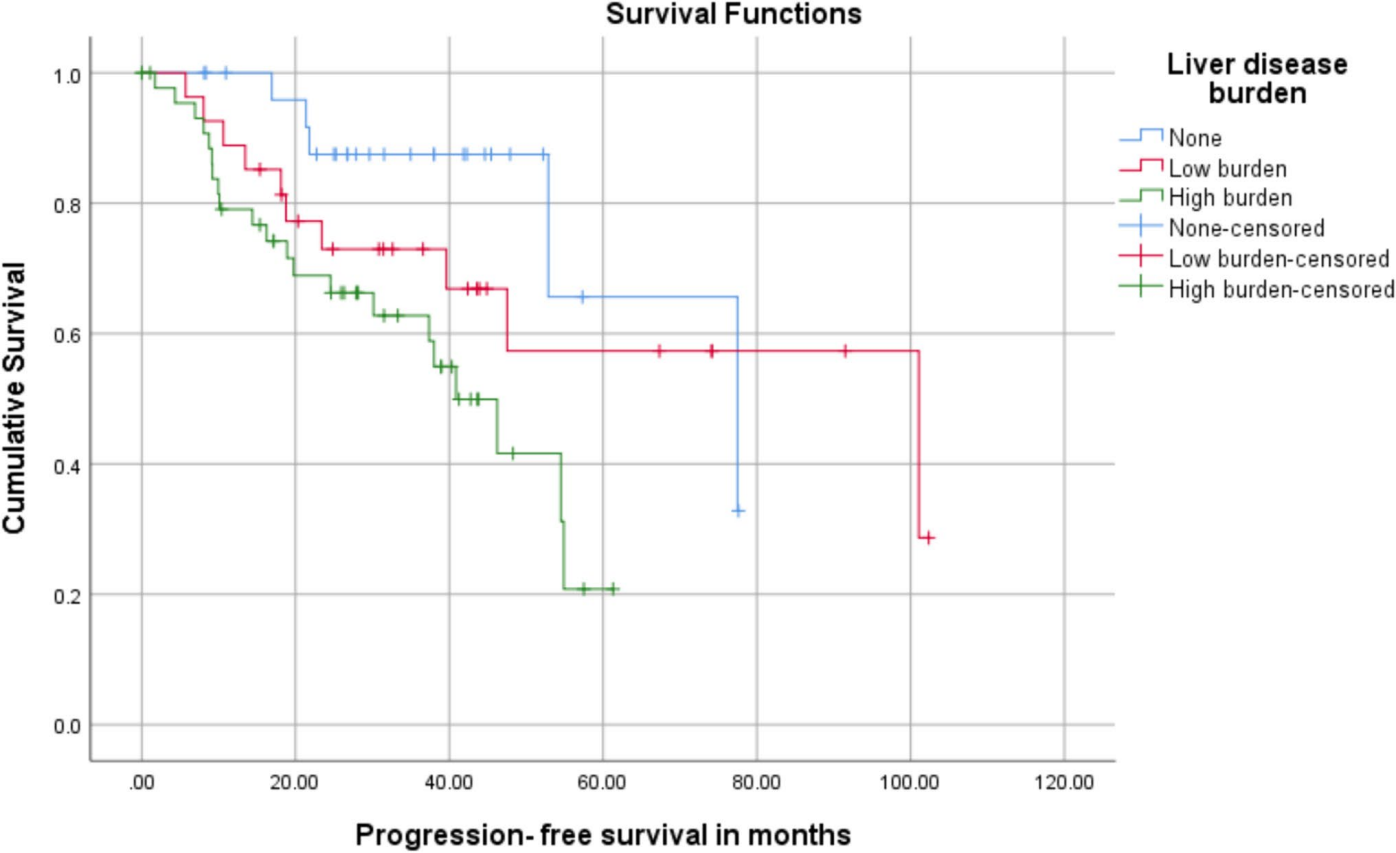
Kaplan–Meier survival curves demonstrating survival figures according to disease burden in the liver. **a** Progression-free survival as per liver disease burden. **b** Overall survival as per liver disease burden

Patients with high-volume liver disease had significantly shorter PFS than patients with low-volume liver disease and no liver metastases (median PFS—40.87 vs. 101.06 vs. 77.47 months; *X*^2^ = 8.094; *p* = 0.017). Estimated 5-year PFS rates of patients with high-volume liver disease, low-volume liver disease, and no liver metastases were 20.8% (SE 0.117), 57.3% (SE 0.12), and 65.6% (SE 0.19), respectively (Table 4 and Fig. 2a). Patients with high-volume liver disease also had a shorter OS than patients with low-volume liver disease and no liver metastases, which trended towards statistical significance but did not meet the pre-specified cut-off value (Fig. 2b, p = 0.053).

**Table 4:** Survival outcomes of patients according to disease burden in the liver

	**Median PFS (in months)**	**95% CI**	**5-year PFS**	**5-year OS**
No liver metastases	77.47	40.44–114.49	65.6% (0.196)	100%
Low-volume liver disease	101.06	23.5–178.61	57.3% (0.123)	96.2% (0.038)
High-volume liver disease	40.87	31.66–50.07	20.8% (117)	75.3% (0.124)

Standard error in parentheses

### Impact of Extra-hepatic Disease on Survival

**Fig. 3:**
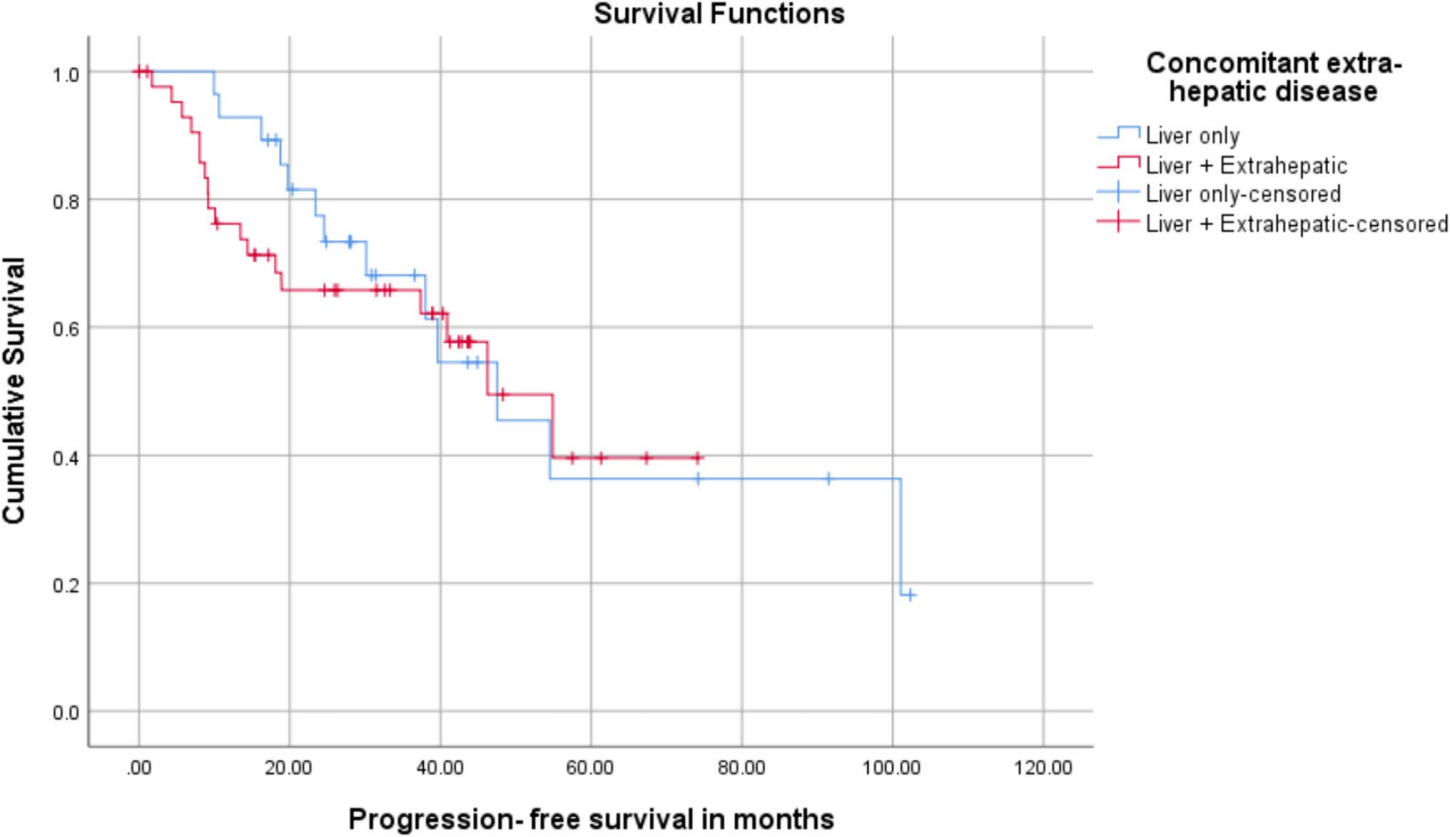
Kaplan–Meier survival curves for progression-free survival of patients according to the presence of concomitant extra-hepatic disease. **a** Progression-free survival and concomitant extra-hepatic disease. **b** Overall survival and concomitant extra-hepatic disease

Patients with liver metastasis were further analyzed for additional sites of metastasis and to assess the impact of additional extra-hepatic disease on survival. There was no difference in the PFS of patients with liver-limited disease compared to those with concomitant extra-hepatic disease (median PFS 47.5 (95% CI 31.11–63.9) months vs. 46.2 (95% CI 30.24–62.21) months, *X*^2^ = 0.189, *p* = 0.664). The 5-year PFS of patients with the liver-limited disease was 36.3% (SE 0.13) as compared to 39.6% (SE 0.122) in patients with concomitant extra-hepatic disease (Fig. 3a). The OS of patients with concomitant liver and extra-hepatic disease, however, was found to be significantly lower than those with liver-limited metastasis (5-year OS 70.7% (SE 0.138) vs. 100%, *X*^2^ = 5.66, *p* = 0.017) (Fig. 3b).

## Discussion

Though previously considered rare tumors, GEP NETs are more frequently encountered in clinical practice, with a documented increase in the incidence across all age groups and primary sites [18]. Managing these so-called ‘indolent’ tumors is complicated and guided by multiple factors, such as the proliferative index, pathology, extent of disease, functionality, and SSTR expression by the tumor cells [4, 5]. While literature is replete with publications with large sample sizes incorporating data from all NET grades, irrespective of the metastatic status, it makes logical sense to start looking at data focusing more on the site, grade, or metastatic status. Since the proliferative index (as ascertained by Ki 67) and the mitotic count are important determinants of tumor aggression, this study addresses prognostic factors in patients with grade 1 GEP-NETS with metastatic disease.

Distant metastases are present in almost half of GEP-NETs at presentation, with the liver being the most common site [19, 20]. The presence of metastases traditionally portends a worse prognosis, but what contributes to a worse outcome in this subgroup of patients is rarely questioned. These patients can be offered a wide range of options, from observation to LAR, PRRT, and radical resections. Selecting one treatment modality over the other is difficult as guidelines are lacking. We hypothesized that if poor prognostic factors could be identified in these patients, it would help select a more aggressive approach where clinically feasible. This may be worth exploring, especially since the baseline tumor grade is low, justifying an aggressive approach. The present study explored this question in three parts—factors affecting survival in metastatic grade 1 GEP-NETs, the impact of tumor burden in the liver and the impact of extra-hepatic disease.

The analyzed PFS in the present study was 54.5 months, with an estimated 3-year PFS of 72.3% and 3-year OS survival of 93.4%. The survival of distant GEP-NETs has been reported in similar figures across international studies. The median overall survival in metastatic GEP-NETs has been recently reported at 34 months (range 32.2–35.8 months) in a study from the United States, analyzing the data of 43,700 GEP-NETs from the SEER (Surveillance Epidemiology and Ends Results) database [19]. These survival figures were irrespective of the primary tumor’s grade and site. The long-term survival of the PROMID trial, published in 2015, reported a median OS of 85 months in metastatic WDNET with a 10-year survival of 45.3% [21]. They also reported better outcomes in patients with low tumor burden in the liver. The PROMID study defined low liver burden as replacing not more than ten per cent of the liver parenchyma by metastasis [22]. The median OS in patients with high liver-tumor burden was considerably shorter (57.5 months vs. 107.6 months, HR = 2.49, 95% CI: 1.36–4.55, *p* = 0.002). The study also emphasized that LAR was more beneficial in patients with a low liver burden than those with a high liver tumor burden. The study also emphasised that patients with low liver tumor burden who died had an increase in liver tumor burden on follow-up scans. The PFS reported in the present study (median PFS—54.5 months) was better than that reported in the PROMID study (14.3 months with LAR and 6 months with placebo) and probably reflects the impact of multiple treatment modalities used [23]. The CLARINET trial also explored the benefit of lanreotide on PFS in metastatic mid-gut NET with Ki 67 < 10% and reported higher PFS than the PROMID study. This benefit was also found irrespective of the liver tumor burden [24]. One important background to this finding is that most of the patients selected for the CLARINET trial had documented non-progression of disease for 3 to 6 months.

This suggests that patients with low liver burden have a better outcome than patients with a high burden. Nevertheless, in patients with low liver burden, an initial close follow-up is warranted; if an increase in disease burden is identified, a more aggressive approach, in the form of PRRT or chemotherapy, may be justified. Peptide receptor radionuclide therapy has been shown to improve PFS in patients with metastatic NET [25]. Chemotherapy or targeted therapy can also be considered per disease burden and progression [26–29]. Surgery offers the best possible cure for NET-liver metastasis where feasible, allowing 5-year and 10-year overall survival in 71–74% and 51–35% of the patients [8, 30, 31]. In such scenarios, the completeness of surgery and the burden of liver disease correlate with disease-free survival [30]. Liver transplantation in selected patients is also a viable option. In a young fit patient, in whom the primary has been resected, with unresectable hepatic metastasis and no extra-hepatic metastatic disease, a possible transplant can be offered [32–34].

Evidence suggests that the current imaging modalities may not be up to the mark to assess disease burden during follow-up, and almost half of the lesions may be missed when comparatively assessed with thin-slice pathological sections [35]. This reflects on the biology of the disease, especially for low-grade tumors, which is also echoed in the present study. A high overall tumor burden affected survival. A high liver tumor load affected PFS. There was also a trend towards a decrease in OS, but it did not reach statistical significance. The presence of concomitant extra-hepatic disease further affected OS as compared to liver-limited metastatic disease.

Most evidence on metastatic grade 1 GEP-NET comes from large international series on large timelines of 2 or 3 decades, with very little evidence segregation for tumor grades, stage and primary site. Focussed data on the tumor grade, stage, and site are required for making meaningful clinical decisions. This, the authors believe, is the biggest strength of the study. The data focuses on grade 1 GEP-NETs, metastatic setting, over a short period, during which the practice of managing this disease is unlikely to have changed much. Moreover, all clinical decisions were protocolised and made after a multidisciplinary discussion. The study can be criticised for its small numbers and retrospective analysis, as reporting and selection biases are unavoidable. The cohort heterogeneity is reflected in the data and may have been difficult to overcome, given the small sample size. Nevertheless, the data was electronically maintained prospectively, patients were followed up using a defined protocol, and all management decisions were made during a multidisciplinary tumor board meeting.

Though properly designed, prospective, controlled trials are the best way to ascertain the impact of more aggressive treatment modalities in high-burden liver-only metastatic grade 1 GEP-NETs, the present study provides meaningful real-world evidence. It can be used as an important roadmap in planning future trials. Moreover, the study emphasises the importance of close follow-up. Aggressive, personalized follow-up is suggested in patients with high tumor burden. Understanding how aggressive treatment regimens will change survival in patients with high-liver burden makes sense. Moreover, follow-up protocols can be relaxed in patients with a low-liver burden when the disease has remained stable for 6–12 months. The understanding that liver tumor burden and the presence of extrahepatic disease affect the progression and overall survival in GEP NETs is well known. Still, the major novel finding of this data is that this also holds for Grade 1 NETs.

In patients with metastatic grade I GEP-NETs, a high liver tumor burden is associated with shorter PFS and decreased OS. The concomitant presence of extra-hepatic metastasis was associated with a further decrease in overall survival.

## Data Availability

The data can be provided by the corresponding author on request.
